# Supplementary material for: Utilizing big data and artificial intelligence to improve the cross-border trade english education
Source: PLoS One. 2025 Nov 5;20(11):e0323941. doi: 10.1371/journal.pone.0323941 (PMC12588483; doi:10.1371/journal.pone.0323941)
Supplement: S1 Data — (DOCX) [file pone.0323941.s001.docx]

**Dataset Description**

This paper presents the AI-based Cross-Border Trade English Education (AI-CTEE) system, which utilizes Long Short-Term Memory (LSTM) networks to create personalized learning experiences, adapt dynamically, and provide real-time language support for trade-related English education. The dataset link is [https://github.com/pavithrachutkie/Trade-English-Education [32](https://github.com/pavithrachutkie/Trade-English-Education%20%5b32)] and contains training data such as Trade English text corpus (CSV), and testing data such as Student Response Data (JSON).
